# Modeling the economic impact for Chile of an import ban on genetically modified maize

**DOI:** 10.1080/21645698.2024.2325180

**Published:** 2024-03-20

**Authors:** William Foster, Jorge Ortega, Gonzalo Vargas

**Affiliations:** a Pontificia Universidad Católica de Chile and Millennium Nucleus Center for the Integrated Development of Territories (CEDIT), Santiago, Chile; b Universidad Técnica Federico Santa María, Santiago, Chile; c Pontificia Universidad Católica de Chile, Santiago, Chile

**Keywords:** Chile, consumer and producer surplus, domestic pork and poultry industries, GM maize import ban

## Abstract

We estimate producer and consumer surplus changes due to a possible GM maize import ban in Chile, which produces only non-GM grains for internal use. Without foreign non-GM sources, the ban’s effect on domestic maize prices would be so significant as to induce Chile to switch from net exporter to net importer of animal products. Fixed factor owners in farm production would benefit significantly, although non-GM maize imports would moderate gains. Total social welfare measures would decline considerably, requiring large offsetting noneconomic benefits for a ban’s political viability. Without non-GM imports, internal maize prices would likely eliminate domestic animal product industries; with possible imports, industries and final consumers would suffer, but much less. Currently, the country is a net importer of grain and a net exporter of pork and poultry, and so most welfare losses on the demand side of the market for maize would be in terms of the economic rents generated by the pork and poultry sectors. International competition would protect final consumers to the extent that animal product imports based on GM feed were permitted.

## 1. Introduction

Although a scientific consensus recognizes the safety, economic potential and benefits of genetically modified organisms,^1,2^ in regulating their use political decision makers around the world have adopted a range of different approaches with varying degrees of restrictiveness. Agricultural exporting countries, such as Argentina, Australia, Brazil, Canada, and the United States, while assessing the safety of GMO products, have less costly approval systems and have approved more GM crops for cultivation. There is a significant number of countries, however, which are relatively more restrictive with respect to the production and use of GM crops, imposing stronger limitations on GM products. These limitations range from labeling regulations to bans on all usage from all sources. Over two dozen countries, including the majority of the European Union, Russia, China and India, have restrictions on the use of this technology, partially or fully banning GMOs.^3,4^ Most allow imports of GM products although restrict internal production. Recently, some countries have intensified restrictions on the use of modern farming techniques, including GM crops, due to perceived possible impacts on health and the environment. In 2021, for example, in addition to restricting GM products, the Sri Lankan government went so far as to attempt a transition to a totally organic agricultural sector, banning imports of synthetic pesticides and fertilizers. This led to severe disruptions in food markets and an associated political crisis.^5^ Currently attracting significant international political attention, going beyond internal production restrictions, the Mexican government announced the gradual implementation of a ban on imports of GM yellow maize and the promotion of traditional production systems.^6^ To date, other Latin American countries, Ecuador, Peru and Venezuela, maintain a ban on GM crop production, while allowing imports.

Of interest in this present study, the Chilean government permits cultivation of GM maize to produce seeds for export and field research,^7^ but domestic GM crops are not grown for national consumption in an environment of ambiguous and incomplete regulations.^8^ All Chile’s maize and soy imports come from GM producing countries. There are periodic political debates regarding either the authorization of GM cultivation for domestic consumption, or, to the contrary, both the elimination of GM seed production and the banning the importation of GM products. As a political matter, this debate is not only fueled by environmentalist interests (e.g., see Greenpeace Chile^9^; see also Greenpeace European Unit^10,11^) but also by domestic producers of import-competing crops who periodically complain about international competition and push protectionist legislation.^1^^1^See, for example, https://www.camara.cl/cms/destacado/2018/09/03/solicitan-medidas-concretas-para-proteger-a-los-agricultores-productores-de-trigo-y-maiz/. Legislators overwhelmingly voted to ask the executive branch to protect wheat and maize farmers and to examine Chile’s free-trade agreement with Argentina, Brazil and other countries (i.e., MERCOSUR). Recently, some representatives in the Chilean national legislature have been seeking to establish large ecological protected areas in which GM organisms are prohibited for seed production and feed in animal production.^12^ In addition to maize consumers in the animal products industry, there are countering biotechnology interests in Chile, which view recent increased interest in restricting GMOs with alarm and are attempting to inform the public regarding what they consider are the potential costs, if Chile were to follow the Mexican precedent (e.g. Chile Bio^13^).

This present study evaluates the annual impacts on traditional economic welfare measures that a possible Chilean ban on importing GM maize would have on domestic producers and users/consumers. As discussed in Qaim,^14^ studies of the welfare effects of the introduction of insect-resistant and herbicide-tolerant crops demonstrate net benefits to producers and consumers, resulting in large aggregate welfare gains, plus benefits to the environment and human health. Furthermore, regulations and unfavorable institutional frameworks reduce potential benefits of GM crops. The analysis in this present paper follows the basic partial-equilibrium approach applied by previous authors (e.g. Falck-Zepeda et al.^15^ particularly the approach of Macall, Kerr and Smyth^6^ to the welfare implications of the Mexican government’s move to ban GM imports. Basic information regarding producer and consumer surplus changes would represent a key contribution to the political decision-making process, which should weigh the economic, environmental, social, and psychological factors linked to national food safety and security.

In the case of Chile, the question of economic welfare as measured by consumer surplus is complicated by the nature of the markets for the final products derived from the use of maize as an input, almost entirely in the manufacture of animal products. And given the scale of meat production relative to internal consumption, and given Chile’s near complete openness to international markets, one should distinguish between maize buyers in the animal products sector and final consumers of those products. In fact, around 80% of the maize consumed in the country is imported, mainly used to produce poultry and pork, milk and eggs.^16^ And more than 80% of maize from any source, domestic or international, is used in the production of only two exported products: chicken and pork, the international shipments of which have grown in recent years to reach 40% of pork production and 20% of poultry.^16^

Knowledgeable analysts of the Chilean meat industry contend that the country’s semiarid, Mediterranean climatic conditions and natural barriers produce a comparative advantage in meat production due to lower costs in maintaining sanitary conditions and to low humidity and moderate temperatures that favor productive efficiency and reduce energy costs.^17-^^19^ Natural comparative advantages, complemented by industry investments in reaching scale economies and establishing international brand reputations and marketing infrastructure, have allowed Chile a notable position as an importer of grain in its raw form and an exporter of grain transformed into meat. Therefore, domestic consumer surplus as measured from the domestic demand for maize would reflect the welfare impacts on the surplus – or economic rent – going to the owners of the “fixed factors of production” in the animal products industry (geography, scale, reputation, etc.) as well as the impact on the surplus going to national final consumers of animal products. To the extent that the owners of fixed factors in animal production are Chilean nationals, this aggregated consumer welfare measure would be informative as to the total domestic economic welfare losses that would result from a complete GM maize ban.^18^

In political-economic terms, however, policy decision makers would likely place different weights on the welfare changes suffered by final consumers, which would be the vast bulk of Chilean society, and the losses to the economic rents of a relatively concentrated class of industry owners. While annual per-capita consumption of maize-based animal products is high in Chile (e.g., in 2021, 35 kg for chicken, 25 kg for pork, 20 kg for beef),^16^ final consumer impacts would be buffered by international arbitrage opportunities. Adjusting for transport and intermediation costs, on the low side, the FOB price for exports represents the opportunity costs of domestic sales facing animal product producers; and on the high side, the CIF price represents the price of imported alternatives to domestic products facing large, domestic intermediaries. These intermediaries are one of the relatively few dominant supermarket chains which can easily access very large-scale suppliers in neighboring countries, mainly Argentina and Brazil. We account for the potential cushioning effect on final consumers of meat due to the access to international markets in evaluating the welfare impacts of a potential GM maize ban.

In the following section, we present the Chilean historical and current context in which a ban on GM maize imports would impact domestic producers and users/consumers (i.e., the animal product industry). The third section describes the basic methodology employed and the econometric approach on which the welfare measurements are based. The fourth section presents the results and interpretation of the estimate models, and the fifth section concludes with the main implications of the study. In general terms, we find that the estimated effects on domestic prices of a GM maize ban are of such magnitude that it would be probable that Chile would go from being a net exporter of animal products to a significant importer of poultry and pork without reasonably priced alternative external sources of non-GM maize. Domestic owners of fixed factors in maize production (mainly landowners) would benefit significantly from a GM maize ban, although the possibility of non-GM maize imports, albeit at elevated prices, would obviously moderate such gains. Total social welfare as measured by producer and consumer surplus would decline considerably; and, if a ban were to be implemented, such losses would have to be balanced with significant offsetting environmental and/or psychological benefits as assessed collectively by participants in the political-economic process. Without non-GM maize imports, the internal price of maize would reach several times their current levels and open the strong possibility that domestic industries reliant on maize would simply disappear, at least in the large-scale, capital-intensive forms in which they currently operate. With possible imports of non-GM maize at a 30% to 50% price premium, both the animal-product industry and final consumers would suffer, but much less. In any case, final consumers would be protected to a degree by international competition, insofar as foreign poultry, pork, and other goods are permitted to enter domestic markets as derivatives of grains produced elsewhere. Final consumers would suffer higher prices and welfare losses to the extent that an anti-GMO policy was to extend to animal products based on GM feed.

## 2. Background: Chilean Maize Production and Use, and Final Meat Consumption

Modern maize production in Chile is primarily aimed at animal consumption. Domestic production for feed is exclusively of non-GM varieties, although an important GM seed-production industry exists exclusively for export. Sweet corn production for direct human consumption is a separate, non-tradeable horticultural market and would remain unaffected by a GM import ban. Data from the Chilean Office for Agrarian Studies and Policy (ODEPA) show that imports now represent four-fifths of internal consumption.^2^^2^Data for internal production, consumption and international trade are taken from the easily accessible data banks maintained by ODEPA: https://www.odepa.gob.cl/estadisticas-del-sector/estadisticas-productivas and https://www.odepa.gob.cl/estadisticas-del-sector/comercio-exterior.Domestic production has not matched the growth in maize demand, which has been driven overwhelmingly by the growth in the production of poultry and pork, but also to a degree by milk, eggs and beef. The chicken and pork industries account for more than 80% of total maize consumption (from domestic and international sources), and our focus is on these industries as representative of the domestic demand for maize. The production of turkeys and other poultry certainly follows the behavior of the chicken sector with respect to prices, and egg production has followed the growth pattern of chicken and pork. Although there are no specific data on maize consumption for egg production, it is a smaller proportion compared to consumption for chicken and pork.^3^^3^If an average conversion rate of 100 grams of maize per egg is considered (Personal communication with Jaime Fernández, Production Manager of Ecoterra), the total consumption of maize would be approximately 0.45 million tons in 2020, which is equivalent to 13% of total maize consumption. In the case of maize for dairy cows, geographically dispersed primarily in southern rainfed and pasture zones, reliable data on maize consumption is unavailable but its proportion of use is considerably lower and much of it in the form of silage.

**Figure 1.**
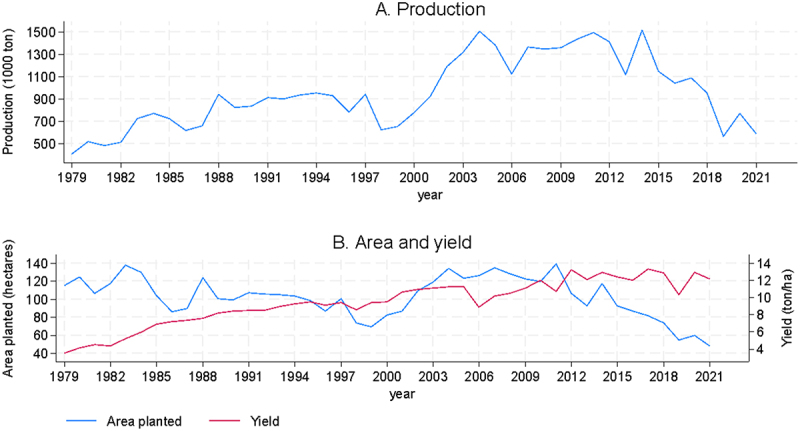
Production, planted area and yield of maize for feed in Chile.

Figure 1 shows the evolution of production, land area and per-hectare yields of maize for animal consumption (i.e., excluding seeds for export) for the period 1979 to 2021. The average area planted in the last 40 years is 103 thousand hectares, although with notable fluctuations over time, driven both by the relative profitability of alternative land uses but mainly by variations in the expected availability of irrigation water, which in turn is driven by total precipitation levels. The largest traditional maize-producing zones have a semi-arid climate (similar to the Central Valley of California) with a rainy season that supplies water to agriculture via aquifers and canals, much of it originating from snowmelt in the Andes. While agricultural productivity per hectare is relatively high, it is nevertheless dependent on irrigation water availability and farmers adjust their planning decisions accordingly. One notes that during the four decades of data, the maximum hectares devoted to maize were 135 and 139 thousand hectares in the years 2007 and 2011. And notably the minimum areas correspond to the recent years of 2020, 2021 and 2022, with only 55, 60 and 48 thousand hectares. The years with the least hectares correspond precisely to the years with the least yearly rainfall. Considering the average of the last decade, a period of relatively low rainfall, the surface area has been 81 thousand hectares, 20% below the longer-term historical average.

Yields have shown a significant upward trend in recent decades. In the late 1970s yields were 4 t/ha (metric tons per hectare) but during the following 20 years new technologies and an increasingly intensive use of inputs led yields to exceed 10 t/ha by the year 2000. The rate of yield increases moderated somewhat and yields now stand at 13 t/ha, among the highest in the world despite the non-GM varieties grown. As seen in Figure 1, the national production of maize shows fluctuations mainly due to variations in area and yields. The years of record total production in Chile correspond to high yields coupled with relatively high total hectares under cultivation. Total hectares have decreased more recently, leading to the current relatively low total production, due in mayor part to limited water resources and the competition with other crops. The geographical distribution of maize planting has changed over time, moving south in response to an increasing water scarcity, competition with other crops more suited to arid conditions, and the relative decline in production costs in areas with less dependence on irrigation.

**Figure 2.**
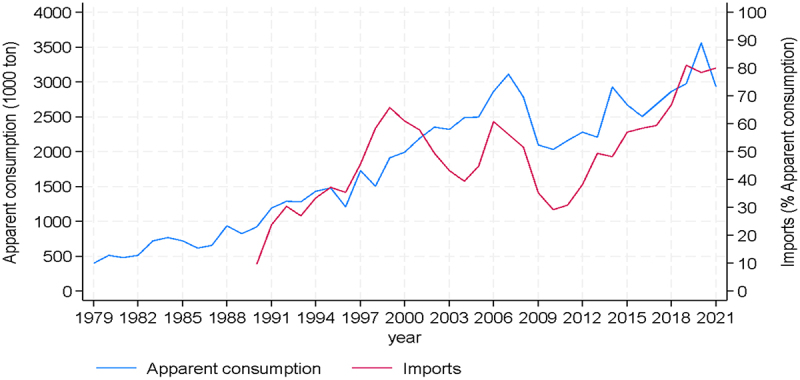
Apparent consumption and imports of maize.

By contrast, as seen in Figure 2, there has been sustained growth in the consumption of maize during the four decades considered in this analysis. There is a practically uninterrupted upward trend until 2008 in maize consumption, when there was a pronounced decline following the economic shock following the so-called subprime financial crisis. Consumption recovered, however, and has reached highs of approximately 3 million tons in recent years. Imports have been the source to satisfy this increasing demand, and in the last three years they represent 80% of total maize use, much higher than the 10% at the beginning of the 90s.

**Figure 3.**
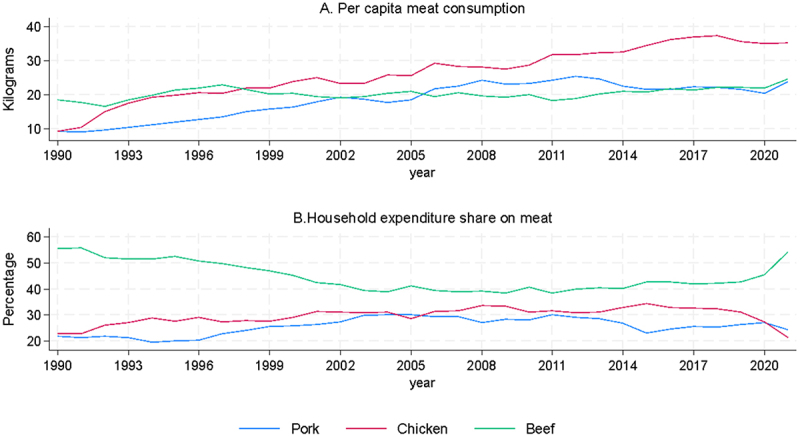
Evolution of apparent meat consumption per capita and expenditure share.

As household incomes have risen with economic development, there has been a notable increase in the consumption of animal protein in Chile. The expansion of the domestic poultry and pork industries (and a growing international market in animal products) has promoted a decline in the relative prices of those meats and a substantial growth in their per capita consumption. As seen in Figure 3, in 1990 the annual consumption of chicken and pork was below 10 kilos per capita, but in recent years the consumption of chicken is greater than 35 kilos and that of pork greater than 25 kilos per capita, a substantial improvement in households’ diets. (Beef has remained relatively stable at around 20 kilos per capita.)

**Figure 4.**
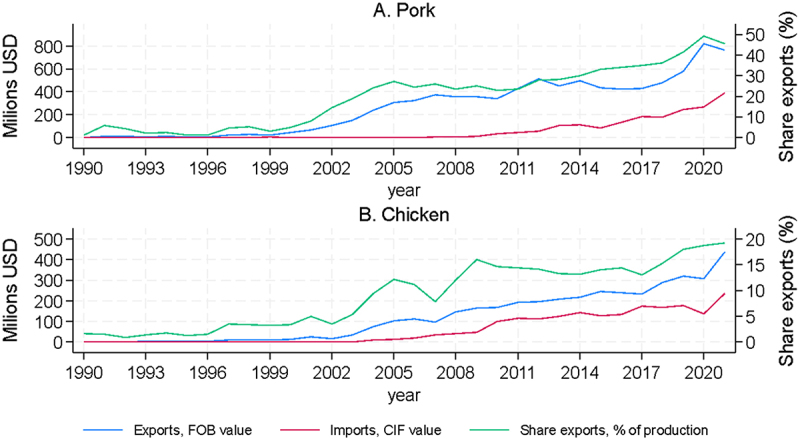
Pork and chicken imports, exports and export share of domestic production.

An increase in scale and the related increase in productivity of the domestic industry have kept imports (typically in bulk frozen form) to a limited proportion of this increase in meat consumption. In fact, the domestic poultry and pork industries are competing as exporters in international markets (see Figure 4), allowing them to reach levels of scale and efficiency beyond those permitted simply by satisfying the domestic market alone. In the early years of the modern poultry and pork sectors (roughly between 1980 and 2000) growth in production was oriented mainly to national consumption, but from the 2000s onwards export destinations became important outlets for rising production. Meat product exports have accelerated in recent years, now exceeding a billion dollars in value per year. Currently, in relation to the total volumes produced in the country, pork exports have reached more than 40% and chicken exports are close to 20% per year. The Chilean poultry and pork industries are unusual to the extent that they are export-oriented sectors that depend fundamentally on imported inputs. The comparative advantage of the country is found in a geography and climate favorable to low production costs and good sanitary conditions for animal health, and in the productive efficiency related to scale, human capital and technological capacities^17–19.^ Poultry and pork are very much globally integrated “value-added” industries, where any increase in basic production costs would significantly affect international competitiveness.

## 3. Methodology

### 3.1. Estimating Welfare Impacts of a GM Maize Import Ban

We estimate the impact on the welfare measures of domestic maize producers and buyers/feed-users for two basic scenarios: the first without non-GM maize imports and the second with non-GM imports but at a higher price that current international maize prices. We also estimate the effect of a GM maize import ban on final domestic consumers of chicken and pork meat.

Several simplifying assumptions are worth noting here. First, we abstract from considerations of risk and uncertainty, focusing on annual expected net income generated in domestic maize production and in the meat industry that demands maize as feed. A Chilean ban on the import of a well-known technology, GM maize, would lead to higher domestic feed costs without introducing the uncertainties sometimes associated with possible irreversible, longer-term benefits or costs, as has been studied in the case of novel technologies.^4^^4^See ^21–24^ Second, other than a ban on GM maize imports, we assume that there are no other policy interventions with respect to other feed sources, such as soy. A ban on GM maize imports will alter the prices paid for maize but not the prices of other inputs, such as soybeans or soymeal, which would still be determined in international markets. The Chilean domestic animal production industry would adjust the quantity demanded of maize and other feed ingredients according to a higher maize price that results from the GM maize import ban. Third, as in most welfare analysis, such as that of Macall, Kerr and Smyth,^6^ we assume that all relevant welfare impacts on domestic maize producers and buyers/users are derivable from the annual domestic maize supply curve and the annual domestic maize demand curve; that is, all annual welfare impacts are recoverable from estimating maize producers’ aggregate marginal cost function and maize buyers’ marginal benefit function and are due to changes in maize prices received and paid. A GM maize import ban would not influence the prices of other production inputs, such as soy, energy, packaging, etc. Fourth, we ignore any trivial non-animal feed uses of imported maize in Chile. And fifth, as will be detailed below, the supply and demand curves are approximated by constant price-elasticity functions.

Finally, the reader should also note that the estimates of welfare changes presented here are annual benefits and losses based on comparative statics applied to estimated yearly supply and demand curves. One can translate these results into cumulative, multi-year impacts of a GM maize impact ban (which would be considerably higher in net present value terms) with the introduction of an appropriate discount rate.

The first scenario analyzed is that of prohibiting imports of transgenic maize but where there are no other sources of non-GM maize except internal production. Self-sufficiency in maize consumption for animal feed would lead to an equilibrium price and quantity determined solely by the intersection of domestic supply and demand. The second scenario assumes that there is an upper limit to the internal price of conventional, non-GM maize given by a potential international market ready to supply Chilean buyers with non-GM maize at a price, say, 30% higher than the current GM international price. We also estimate welfare effects of having a GM ban but with the international price of non-GM maize being 20%, 40% and 50% higher than the current price.

### 3.2. The Basic Model

**Figure 5.**
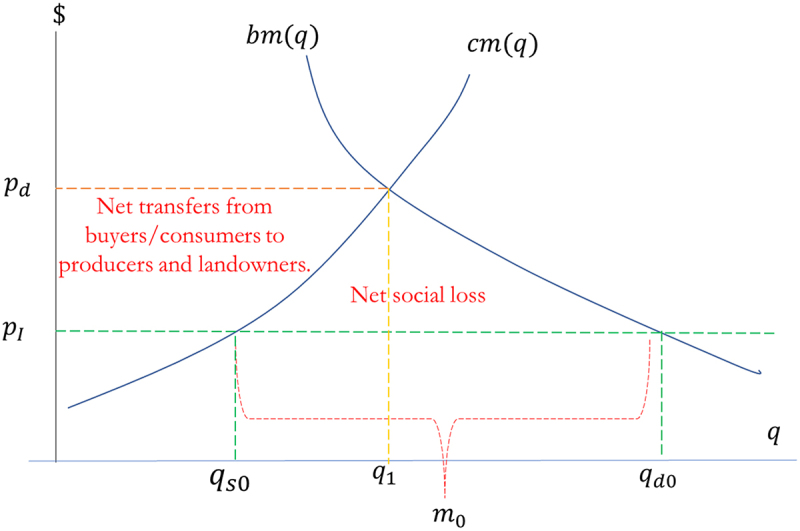
Basic model of the maize market.

Source: authors’ elaboration

The basic model to estimate the impacts of the import ban on transgenic maize begins by defining the interest groups that will be impacted by the import ban policy: (i) Chilean landowners and farmers of conventional, non-GM maize, (ii) buyers/users of maize for feed in the animal products industry, and (iii) final consumers of food products (e.g., consumers of pork, chicken, etc.). One can estimate both producer and consumer surplus from information in the domestic market for maize in the form of the elasticities of demand and supply and two reference points with respect to the quantities domestically produced and total purchases in a year. Figure 5 shows the basic model in the case of no other sources of non-GM grain. The supply of domestic production represents the marginal opportunity costs of production, $\mathrm{mc}\left(q\right)$, determined by the potential alternative uses of resources applied to maize production (land, water, labor, etc.). The domestic demand for maize represents the marginal benefits of the grain, $\mathrm{mb}\left(q\right)$, determined by domestic and export sales of animal products derived from the input. The international price of maize, $p_{I}$, represents the perfectly elastic supply of imported maize, given that Chile represents a very small share of the quantities globally demanded of the grain.

The change in producer surplus, ∆PS (a gain in economic rent), associated with an *increase* in quantity sold from $q_{s0}$ to $q_{s1}$ and an increase in domestic price from $p_{0}$ to $p_{1}$, is the difference between gross receipts after and before the ban, minus the increase in variable costs due to an expansion in maize production:

(1) $$\Delta \mathrm{PS}=p_{1}q_{s1}-p_{0}q_{s0}-\overset{q_{s1}}{\underset{q_{s0}}{\int }}\mathrm{mc}\left(q\right)\mathrm{dq}$$

The change in consumer surplus, ∆CS (a loss), associated with a *decrease* in quantity purchased from $q_{d0}$ to $q_{d1}$ and the increase in price from $p_{0}$ to $p_{1}$, is the difference between the total spending on maize before and after the ban, plus the loss of gross benefits due to the reduction in the use of the input in the manufacture of animal products:

(2) $$\Delta \mathrm{CS}=p_{0}q_{s0}-p_{1}q_{d1}-\overset{q_{d0}}{\underset{q_{d1}}{\int }}\mathrm{mb}\left(z\right)\mathrm{dz}$$

The net social loss is the sum of the two surplus measures one positive, the other negative: ΔPS+ΔCS.

As a practical computational matter, in this present analysis the change in producer surplus due to a change in price from $p_{0}$ to $p_{1}$ is derived from an estimated constant-price-elasticity supply function of the form $q=Ap^{\varepsilon }$, where ε is the (constant and positive) elasticity of supply with respect to maize price received. For the change in producer surplus, one integrates the supply curve between the two prices: $\Delta \mathrm{PS}=\overset{p_{1}}{\underset{p_{0}}{\int }}Ap^{\varepsilon }\mathrm{dp}=p_{0}q_{s0}\left[{\left(\frac{p_{1}}{p_{0}}\right)}^{1+\varepsilon }-1\right]{\left(1+\varepsilon \right)}^{-1}$. Similarly, the change in consumer surplus is derived from an estimated constant-price-elasticity demand function of the form $q=Bp^{\eta }$, where η is the (constant and negative) elasticity of demand with respect to maize price paid. For the change in consumer surplus, one integrates the demand: $\Delta \mathrm{CS}=\overset{p_{1}}{\underset{p_{0}}{\int }}Bp^{\eta }\mathrm{dp}=p_{0}q_{d0}\left[{\left(\frac{p_{1}}{p_{0}}\right)}^{1+\eta }-1\right]{\left(1+\eta \right)}^{-1}$. Note that, in addition to estimating the supply and demand elasticities, the two surplus changes depend on three values: the post-ban maize domestic maize price relative to the pre-ban price, $p_{1}/p_{0}$, the initial revenues earned by domestic producers, $p_{0}q_{s0}$, and the initial total expenditures on maize by buyers, $p_{0}q_{d0}$.

To calculate producer and consumer surplus changes, we first find the implied equilibrium price, $p_{d}$, that would result from the hypothesized ban on GM imports and the equilibrium quantity produced and consumed, $q_{1}$, at that price (given by the intersection of the internal supply and demand curves).^5^^5^In the case of the constant price elasticities of supply and demand used in the present study, in equilibrium without imports, $Ap_{d}^{\varepsilon }=Bp_{d}^{\eta }$, which implies $p_{d}={\left(\frac{B}{A}\right)}^{\frac{1}{\varepsilon -\eta }}$ and $q_{1}=A^{\frac{-\eta }{\varepsilon -\eta }}B^{\frac{\varepsilon }{\varepsilon -\eta }}$. We use the estimated supply elasticity, ε, and demand elasticity, η, to extrapolate the supply and demand curves as a first-order approximation of the impact of the import ban. The equilibrium price under the import ban, $p_{d}$, is a multiple of the current international price, $p_{I}$:

(3) $$p_{d}=p_{I}{\left(\frac{q_{d0}}{q_{s0}}\right)}^{\frac{1}{\varepsilon -\eta }}$$

The multiplier factor is determined both by the degree to which current domestic consumption, $q_{d0}$, exceeds current national production, $q_{s0}$, and by the magnitudes of the elasticities of supply and demand. The less sensitive the supply and demand curves are, the higher the new equilibrium price will be compared to the current international price. The new equilibrium quantity, $q_{1}$, is also calculated from the original levels of domestic production and consumption and the elasticities of supply and demand:

(4) $$q_{1}=q_{d0}^{\frac{\varepsilon }{\varepsilon -\eta }}q_{s0}^{\frac{-\eta }{\varepsilon -\eta }}$$

The changes in producer and consumer surpluses are calculated using both the new equilibrium price and quantity. The post-ban maize domestic maize price depends on the specific scenario under examination, as discussed in the following paragraphs.

### 3.3. Scenario 1: A GMO Ban without Imports of Conventional Maize

The current level of domestic production, $q_{s0}$, establishes the starting point from which domestic supply would increase to replace a fraction of lost imports. The current level of total domestic purchases of maize, $q_{d0}$, is the starting point from which domestic consumption would decline in response to an increase in internal price from the current internationally determined price, $p_{I}$, due to the inability to access traditional foreign suppliers. Initially, given current market conditions and regulations, domestic quantity demanded exceeds domestic production leading to a level of imports, $m_{0}=q_{d0}-q_{s0}$ (which in the case of Chile is approximately 80% of total consumption). With a ban on GM maize imports, and in the absence of imports, price would increase to an internally determined level, $p_{d}$, that would equilibrate the quantity domestically produced and consumed, $q_{1}$. The exact level of price and quantity that would result from a ban would depend on the estimated elasticities of both supply and demand curves.

The area under the marginal cost curve, between 0 and some level of domestic sales, represents the total variable costs of domestic maize production. Gross receipts (quantity produced times the sales price) less production costs represent the “producer surplus” earned by owners of limited resources (usually land or special talent in maize production). The area under the marginal benefit curve, between 0 and some level of purchases, represents the gross consumer benefits from using the good. Gross benefits minus revenues earned by producers represents the “consumer surplus.” Note that the international price, $p_{I}$, represents the social opportunity cost for Chile as a whole and is the equilibrium price for domestic buyers and producers without a prohibition on importing maize. As with any trade restriction that places a wedge between domestic and international prices of a good, there are social losses due both to over-production and to under-consumption of the good. By prohibiting maize imports and without international substitutes, imports fall to zero, and the new equilibrium price, $p_{d}$, would increase to induce an inflow of resources from other activities reallocated to domestic maize, causing an efficiency loss to Chile in the aggregate. Buyers of maize would lose welfare via a higher price of the product; and, being induced to reduce maize use, there is a reallocation of resources from meat production and final meat consumption to other goods and services, causing an additional efficiency loss to society on the demand side.

### 3.4. Scenario 2: A GMO Ban with Imports of Conventional Maize

With the possibility of importing conventional, non-GM maize, however, the social losses would be mitigated. Although there are some countries where the cultivation of GM maize is not allowed (such as in the European Union, where only Spain and Portugal grow GM maize), currently there are no significant, large-scale exports of certified non-GM maize. In general terms, the most internationally competitive maize producers use GM technologies in more than 90% of their total production. Without an international market of sufficient depth for conventional maize, this almost exclusive emphasis on GM maize trade will likely continue. The immediate obstacles to the development of parallel GM and non-GM maize markets are the lack of traceability systems and/or certifications that would allow sellers from countries of origin to guarantee the production, storage, and transportation of non-GM grain. Evidently, until today the transaction volumes of a potentially differentiated non-GM product have not been sufficient to cover the fixed costs necessary to establish an integrated trading system and sustain the associated futures and derivatives markets that would allow planning and managing risks linked to climatic and political disruptions.

Notwithstanding the current absence of significant international trade in non-GM maize, one finds news reports that the Mexican government is looking to contracts with external producers in Argentina, Brazil and the United States to meet “normal” quantities of internal demand that could not be met by increasing domestic production.^6^^6^See, for example, “Mexico plans to buy non-GMO corn from the U.S., other countries as it moves ahead with GMO ban” by ^25^ A hypothetical scenario is not unreasonable that companies that use grain in Chile could also sign similar production contracts for conventional maize. In addition to the higher production costs per ton of non-GM maize, it would be necessary to establish a dedicated organization for the signing and supervision of these contracts and subsequently ensure that the products are not mixed with GM products in storage and transport. According to consultations with industry participants, a system of this nature could represent an increase in CIF prices on the order of 30% higher than current prices.

**Figure 6.**
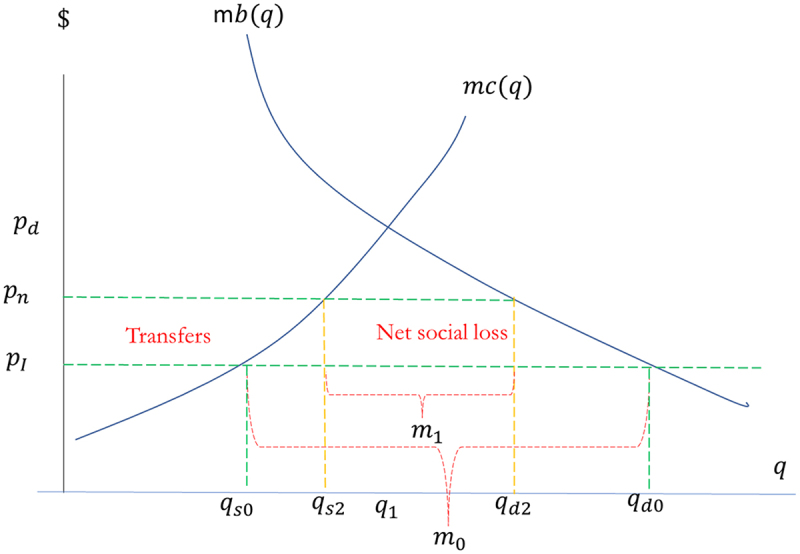
Impact of increased import costs of grain maize.

Source: authors’ elaboration

As seen in Figure 6, a new import price, $p_{n}$, of non-GM maize would be between the current import price and the equilibrium price in the absence of any imports. Internal production would increase from current production to $q_{s2}$, the quantity consumed would decline to $q_{d2}$, and imports would decline from $m_{0}$ to $m_{1}$. Again, although mitigated, there would be an increase in producer surplus, a decrease in consumer surplus, and a social loss resulting from misallocated resources to production and lower consumption.

### 3.5. Measuring the Impact on Final Consumers of Chicken and Pork

**Figure 7.**
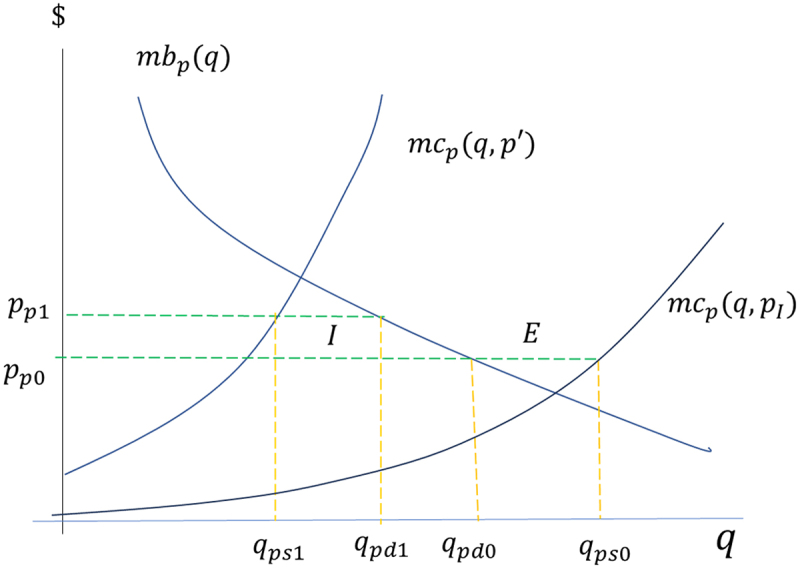
Basic model of the animal products market (pork as an example).

Source: authors’ elaboration

Given the level of exports of poultry and pork, the production of which represents more than 80% of total maize consumption, of additional interest is the impact on the welfare of domestic final consumers. To illustrate, consider the case of the domestic pork market as shown in Figure 7. The initial pork supply curve, $mc_{p}\left(q,p_{I}\right)$, represents the marginal costs of national production given the international price of maize, $p_{I}$. The supply curve after the ban, $mc_{p}\left(q,p{\;}^{\prime }\right)$, represents the marginal costs of pork production given the price of maize that results from the ban on GM imports, $p{\;}^{\prime }$, either determined in equilibrium in the domestic market, $p{\;}^{\prime }=p_{d}$, or equal to the import price of non-GM maize, $p{\;}^{\prime }=p_{n}$. The demand curve for pork, $mb_{p}\left(q\right)$, represents the marginal benefits curve to final consumers. Currently, Chilean producers are net exporters of pork and chicken, so the reference price to consumers would be set by the FOB price of pork exports, $p_{\mathrm{pI}}$. At this price domestic pork consumption would be $q_{\mathrm{pd}}$ and domestic pork production would be $q_{\mathrm{ps}}$, resulting in exports, $E=q_{\mathrm{ps}0}-q_{\mathrm{pd}0}$ in Figure 7.

A ban on GM maize would increase the marginal cost curve of pork production, probably (as our estimates below indicate) to such a degree that the country would become a net importer of both pork and chicken without a foreign source of non-GM grain. And if imported products are close substitutes for domestic products, then final consumers would be indifferent to the change except to the extent that product prices would increase due to the shift from the FOB price (as the reference for exporting meat by domestic producers) to the higher CIF price (as the reference for importing supermarkets and other intermediaries). Net imports are represented by $I=q_{\mathrm{pd}1}-q_{\mathrm{ps}1}$ in Figure 7. We estimate an impact on final consumer welfare as the change in consumer surplus at the two reference prices, before and after the ban, which can be approximated by the change in total consumer expenditures based on current quantities consumed. The final consumer is protected to a certain extent from the effects of a GM maize ban by the possibility of accessing imported poultry and pork. As is shown below, the harmful effect of the ban concentrates on the users of maize in the animal products industry, especially pork and chicken producers.

Numerical values for these surplus measures are based on the estimation of domestic supply and demand curves using recent historical observations of quantities supplied by domestic producers, total quantities demanded by domestic meat producers, and international prices.

### 3.5. Empirical Estimation of Maize Supply and Demand Elasticities

To calculate the elasticities of the supply of maize for consumption (excluding seed and other minor uses), a basic linear regression model in logarithms is employed:

(5) $$\ln\left(q_{t}\right)=\alpha_{0}+\alpha_{1}\ln\left(p_{t}\right)+\alpha_{2}\ln\left(pp_{t}\right)+\alpha_{3}\ln{\left(pp_{t}\right)}^{2}+\alpha_{4}t_{t}+\alpha_{5}t_{t}^{2}+u_{t}$$

where *q*_*t*_ is the production of maize in metric tons, *p*_*t*_ is the price of maize (we use the FOB US$/ton averaging the no. 2 yellow price US Gulf and Buenos Aires), *pp*_*t*_ is precipitation in millimeters (averaged over four production regions), *t*_*t*_ is a trend variable to capture technological change and *u*_*t*_ is the error term. The parameter *α*_*1*_ measures the price elasticity of supply ($\frac{\partial \ln q_{t}}{\partial \ln p_{t}}$) and the parameters *α*_*2*_ to *α*_*5*_ measure the response elasticities to the other variables. As an alternative approach to estimating the supply elasticity, we also apply the log-linear model to cultivated hectares as the dependent variable. A separate analysis of per-hectare yields shows that they tend to fall as hectares increase, due to soils less apt to maize being brought into production; but, at least over the period for which data are available, per-hectare yields are insensitive to price fluctuations once one controls for total hectares, climate variables and simple trends to account for technological changes. The estimated supply elasticity using this alternative hectare-yield approach is recovered by summing the price elasticity for hectarage response plus the associated contribution of yield changes brought about shifts in hectares planted.^7^^7^Defining total production, $q_{t}$, as the product of hectares, $H_{t}$, and yields, $y_{t}$, one notes that $\ln q_{t}=\ln H_{t}+\ln y_{t}$, and so, with yields being sensitive to scale but not to price, the supply elasticity would be $\frac{\partial \ln q_{t}}{\partial \ln p_{t}}=\frac{\partial \ln H_{t}}{\partial \ln p_{t}}+\frac{\partial \ln y_{t}}{\partial \ln H_{t}}\frac{\partial \ln H_{t}}{\partial \ln p_{t}}$. The supply elasticity calculated in this form is (unsurprisingly) similar to that of the simple approach using total production. As another robustness check, we also estimate the national supply relationship in Equation (1) for four main maize producing regions where production data are available. (We refer to these regions in the results in Table 1 as O’Higgins, Maule, BioBio and Metropolitan.) Using these disaggregated results, the relevant national elasticity of supply would be the weighted average of the regional supply elasticities. We find that the national-level supply elasticity varies little between the two approaches.

**Table 1.** Price elasticities of the supply of maize for animal feed.^a^

	National	O’Higgins	Maule	BioBio	Metropolitan	National aggregated from regional^d^
Aggregate production	0.379***	0.080	0.965***	1.079***	0.705***	0.376***
	(0.113, 0.646)	(−0.177, 0.337)	(0.583, 1.347)	(0.536, 1.622)	(0.256, 1.154)	(0.093, 0.753)
Hectares	0.473***	0.124	0.883***	0.799***	0.622***	–
	(0.222, 0.724)	(−0.095, 0.344)	(0.500, 1.266)	(0.409, 1.189)	(0.219, 1.024)	
Indirect yield effect^b^	−0.116*	0.007	0.012	0.152	0.131*	–
	(−0.236, 0.004)	(−0.026, 0.040)	(−0.082, 0.106)	(−0.058, 0.359)	(0.013, 0.250)	
Composite production^c^	0.353**	0.152	0.895***	0.927***	0.742***	0.353***
	(0.152, 0.554)	(−0.077, 0.381)	(0.529, 1.261)	(0.455, 1.399)	(0.294, 1.191)	(0.138, 0.747)

(a) Estimates are from Tables A1–A5 in the Appendix. 95% confidence intervals in parentheses. National estimation of production and area by Ordinary Least Squares with Newey-West standard errors robust to heteroscedasticity and autocorrelation (HAC); national per hectare yield estimation with instrumental variables using Generalized Method of Moments and HAC (log FOB price as log surface instrument); regional estimates by System of Apparently Unrelated Equations (SUR) method; (b) indirect per hectare yield price elasticity calculated as the elasticity of yield with respect to hectares times the elasticity of hectares with respect to price; c/composite production-price elasticity calculated as hectare-price elasticity plus the indirect yield-price elasticity ($\frac{\partial \ln q_{t}}{\partial \ln p_{t}}=\frac{\partial lnH_{t}}{\partial \ln p_{t}}+\frac{\partial \ln y_{t}}{\partial \ln H_{t}}\frac{\partial \ln H_{t}}{\partial \ln p_{t}}$). Asterisks indicate p-values of the estimates: *** *p* < .01, ** *p* < .05, * *p* < .1. d/The national aggregated supply elasticities are derived from the regional elasticities by summing the individual regional elasticities weighted by the share of each region’s production level evaluated at the sample averages. In this exercise, we use the point estimates of the elasticities except for the O’Higgins region where the elasticity is set to zero.

To calculate the elasticity of demand for maize for consumption by the meat producing industry, a similar linear regression model in logarithms was used:

(6) $$\ln\left(c_{t}\right)=\beta_{0}+\beta_{1}\ln\left(p_{t}\right)+\beta_{2}\ln\left(\mathrm{pc}c_{t}\right)+\beta_{3}\ln\left(\mathrm{pc}p_{t}\right)+\beta_{4}t_{t}+u_{t}$$

where *c*_*t*_ is the (apparent) consumption of maize in tons, *pcc*_*t*_ and *pcp*_*t*_ represent the US$/kg FOB prices of chicken and pork, *t*_*t*_ is a trend variable to capture improvements in productivity, the trend of final-consumer preferences toward poultry and pork, and the overall growth in export markets for meats. The term *u*_*t*_ is the error term. The apparent consumption of maize is calculated as domestic production plus imports. The parameter *β*_*1*_ measures the price elasticity of demand and the parameters *β*_*2*_ to *β*_*4*_ measure the response elasticities to the other variables.

## 4. Results

### 4.1. Estimated Supply and Demand Elasticities for Maize

For this analysis, we use historical data from 1979 to 2021 on the yearly hectarage and yields of domestic maize production, the levels of exports and imports of meats and maize, and international prices of relevant products. Data for production and imports of maize and for international trade in meat products derive from information provided by Chilean Ministry of Agriculture (see^16^). Additionally, from the Central Bank of Chile and the World Bank we take GDP per capita, population statistics, exchange rates and international raw material prices.^8^^8^Chilean Central Bank data can be found at https://www.bcentral.cl/inicio, and https://si3.bcentral.cl/siete; World Bank data can be found at https://datatopics.worldbank.org/world-development-indicators/. Historical rainfall data relevant to domestic maize-producing regions were obtained from the World Bank’s climate change knowledge portal.^9^^9^Climate data are found at https://climateknowledgeportal.worldbank.org/.
We present various tables of econometric results in the attached Appendix showing the results of different models to estimate the supply and demand elasticities. Tables A1 and A2 present the regression results for the aggregate, own-price supply elasticity of maize, correcting for autocorrelation and potential endogeneity problems of some of the explanatory variables. Tables A3–A5 present similar regression results but disaggregated at the level of Chile's four maize production regions, from which we then aggregate the regional elasticities to derive a country-level own-price price elasticity.

The important results with respect to elasticities are robust across various specifications. Table 1 summarizes the supply elasticities calculated from the econometric models. Our focus is on a point price elasticity of production at the national level. Using the aggregate national production approach, we find a price elasticity point estimate of 0.38, with a 95% confidence interval between 0.11 and 0.65. Of tangential interest, we find that there is high regional variation in supply sensitivity to prices. One region, O’Higgins, is not sensitive to price, while the other regions have greater elasticities, varying between 0.70 and 1.08. Composite production elasticities, calculated from area and yield, are of similar value to those estimated from total production. The national elasticity aggregated from the regional estimates are similar to that estimated directly from national production. In Table 2 we show the results of the estimates of the demand for maize. (As robustness checks, we also estimated alternative several models, not shown here, all of which produce similar results.) The point estimate of the price elasticity of demand that we use below to evaluate the welfare effects is −0.48, with a 95% confidence interval varying between −0.57 and −0.39.

**Table 2.** Demand model: log apparent maize consumption for feed.

	(1)	(2)
VARIABLES	HAC	GLSR
log maize price, FOB	−0.480***	−0.430***
	(−0.571, −0.388)	(−0.577, −0.283)
log pork price FOB	0.438***	0.321***
	(0.212, 0.664)	(0.093, 0.550)
log chicken price FOB	0.461**	0.436**
	(0.102, 0.820)	(0.067, 0.804)
Trend	0.017*	0.017*
	(−0.003, 0.036)	(−0.003, 0.036)
Constant	9.625***	10.485***
	(6.120, 13.130)	(7.104, 13.867)
Observations	32	31
R-squared	0.903	0.823

95% confidence intervals in parentheses, *** *p* < .01, ** *p* < .05, * *p* < .1. HAC = Newey-West standard errors robust to heteroscedasticity and autocorrelation; GLSR = Generalized Least Squares (Cochrane-Orcutt) with robust standard errors.

### 4.2. Welfare Impacts in the Case of Self-Sufficiency: No Imports of Non-GM Maize

Calculations of welfare impacts are based on current levels of domestic production and consumption ($q_{s0}$ and $q_{d0}$ in Figures 5 and 6), and on point estimates of the price elasticities of domestic supply and derived demand for maize (0.38 and −0.47). To calculate producer and consumer surplus changes, we employ Equations (3) and (Equation 4) to find the implied equilibrium price, $p_{d}$, that would result from the hypothesized ban on GM imports and the equilibrium quantity produced and consumed, $q_{1}$, at that price (given by the intersection of the internal supply and demand curves). We use the estimated supply elasticity, ε, and demand elasticity, η, to extrapolate the supply and demand curves as a first-order approximation of the impact of the import ban.

The changes in producer and consumer surpluses are calculated using both the new equilibrium price and quantity. As reference points, the recent average levels of national production, $q_{s0}$, is 800,000 tons, and the level of apparent consumption, $q_{d0}$, is 3,000,000 tons. Based on these parameters, Table 3 shows the estimated impacts on producer and consumer welfare. Under a scenario of zero imports of non-GM maize, prohibiting imports of GM grain would imply an increase in internal prices of 350%, moving from the current average of US$250/ton to a value of US$1,122/ton. This would imply an increase in producer surplus, ΔPS, by US$1,006 million per year. The equilibrium quantity produced would rise to 1,415 million tons, that is, 76% more than the current domestic production base. In terms of cultivated area, this would reach close to 110 thousand hectares; that is, in the upper range of land devoted to maize in recent decades. (One implication of this estimated increase in production is that the additional land devoted to maize would come at the expense of other crops.)

**Table 3.** Welfare impact in Chile of GM maize import ban under self-sufficiency.

Variable	Impacts
Increase in domestic maize price (US$/ton)	250 to 1,122 (+350%)
Increase in domestic maize produced (1000 tons)	800 to 1,415 (+76%)
Reduction maize consumed (millions of tons)	3 to 1.42 (−53%)
Increase in producer surplus (US$ million/year)	1,006
Decrease in consumer surplus (US$ million/year)	1,678
Net loss in social surplus (US$ million/year)	672

Source: Authors’ calculation based on supply and demand elasticity estimates.

Apparent maize consumption would fall by 53%, compared to the current 3 million tons. The consumer surplus would decline by US$1,678 million per year, 60% more than the gain in producer surplus. The maize consumers would use the input at the level of the mid-1990s, when the animal products sector was beginning its transformation in scale and export orientation. Note that the yearly total loss in social surplus would be US$672 million. The impact of a possible ban on GM maize would be a shock that would produce a profound structural change in the markets for maize and animal products. One can interpret our assumptions as conservative in the sense that maintaining a constant demand elasticity, despite an order of magnitude change in price, implies that meat product industries would continue operating albeit at half scale. Such a change could lead, however, to disruptions in the structure of the industry, if not bankruptcies and closures. In fact, our estimates under this self-sufficiency scenario implicitly assumes that the industry could pass on an important part of the increase in costs to final consumers, which, considering the magnitude of the price rise, could only occur in a product market closed to imports. If the country were to remain open to imports of meat, dairy products and other products, it would be difficult for the local industry to compete. If it did survive, the national meat products industry would regress to volumes observed decades ago.

### 4.3. Welfare Impacts in the Case of Possible Non-GM Imports

Chile, however, is well open to international trade, with free trade agreements with all of its major suppliers of imported grains and meat products. The magnitude of the impact of a GM maize ban would be considerably reduced if maize users were to have some alternative to the domestic input, although at a higher cost. We therefore estimate the changes to producer and consumer welfare for increases in the price of imports of 20%, 30%, 40% and 50% compared to the current average. As seen in Table 4, taking as a reference point the initial production of 800 thousand tons and apparent consumption of 3 million tons, with an increase in the import cost of 30%, internal production would grow by 10%, reaching 883 thousand tons, and apparent consumption would decrease by 12.3%, reaching 2.63 million tons. In this case, the surplus of domestic maize producers would increase by US$63 million per year, and the surplus of buyers/consumers would decrease by US$210 million per year. The annual net social loss would be US$147 million. If the import price were to increase by 50%, production would grow by 16.6%, reaching 933 thousand tons, and apparent consumption would decrease by 18.3%. Producers’ surplus would increase by US$108 million per year, and buyers’ surplus would decrease by US$337 million per year. The annual net social loss would be US$228 million.

**Table 4.** Impacts on welfare under scenario 2: importing non-GM maize with price increases of 20%, 30%, 40% and 50%.

Variable	Increase in import price from US$250			
	20%	30%	40%	50%
Maize price	300	325	350	375
Increase maize produced (thousands of tons)	800 to 857 (+7,2%)	800 to 883 (+10,5%)	800 to 909 (+13,6%)	800 to 933 (16,7%)
Reduction in maize consumption (millions of tons)	3 to 2.74 (−8,7%)	3 to 2.63 (−12,3%)	3 to 2.54 (−15,5%)	3 to 2.45 (−18,4%)
Gain in producer surplus (US$ million/year)	41	63	85	108
Loss in consumer surplus (US$ million/year)	143	210	275	337
Loss in net social surplus (US$ million/year)	102	147	190	228

Source: Authors’ calculation.

### 4.4. Welfare Effects on Domestic Final Consumers of Chicken and Pork

Unlike the United States and other countries, where there are observable contracts and transactions between animal growers and processors and wholesalers, a branded Chilean chicken or pork product is delivered to supermarket shelves by a single corporate entity vertically integrated all along the production chain. Moreover, there are only four notable brands, of which two dominate supermarket shelf space. The pass-through of maize price to final consumer prices is, therefore, not a statistic easily directly calculable from officially available statistics. Nevertheless, the Chilean poultry and pork sectors are similar to those found in the northern hemisphere in terms of genetics, grower and processor technologies, marketing chain logistics, and retail sectors. One can, therefore, estimate the pass-through from information from local experts familiar with the technical side of poultry and hog production and from available information regarding the farm-to-retail cost structures in countries such as the United States. From discussions with local experts, we estimate that, although the relative weight of maize in the cost structure of live poultry and hog production fluctuates, it currently stands between 30% and 40% of total costs.

The “farm share” of the retail value of fresh meat retail products – that is, the share of the final consumer price attributable to the live animal production stage of the marketing chain – ranges from 30% to 50%.^20^ A 50% increase in maize price, therefore, would imply an approximate increase in the cost of the final product on the supermarket shelf on the order of 5% to 10%. This retail price rise would likely not trigger a large-scale shift to imported animal products. But certainly, a rise of the internal maize price that would accompany a GM import ban without an international market in non-GM maize would lift domestic prices to the degree that intermediaries would access foreign meat suppliers. In a scenario with a ban on GM imports but without an international source of non-GM substitutes, national apparent consumption of maize would fall to approximately half of current levels. As noted previously, if the markets for products derived from maize – such as chicken and pork – were also closed to international trade, the above would mean a substantial drop in the production and consumption of these goods, and a substantial increase in domestic prices.

Chile, however, can import food without major barriers except those related to transport and intermediary costs. Current international treaties would also prevent establishing arbitrary restrictions on imports of animal products although produced from GM feed in their country of origin. There is no evidence that meat from animals fed with GM grain makes any difference for human consumption, and so a ban or limitation of meat imports would be interpreted as a protectionist, non-tariff barrier. Consequently, unlike the domestic maize-using animal products industry, Chilean consumers would be affected to a lesser extent, due to the possibility of importing pork and poultry meat (which would most likely have been produced with transgenic maize in their countries of origin). With the ban on importing transgenic maize, and the consequent substantial increase in production costs without an internationally available non-GM substitute, the prices of domestic pork and chicken would increase substantially. Large market players (large supermarket chains or even some current meat producers who have already-established marketing networks) would increasingly meet final consumer demand through imports.

Consumers of final products would lose to the extent that imports are more expensive than domestic production. To estimate an upper limit to the welfare impact on final consumers, we simply consider the increase in total expenditures due to an increase in retail product prices in the event of a shift from the case of Chile as exporter of chicken and pork to the case of consumer dependence on imports. We estimate this final consumer cost increase using a first quarter 2023 price of pork of US$4.71/kg and of chicken of US$3.53/kg,^10^^10^We also take a first quarter 2023 approximate exchange rate of 850 Chilean pesos per dollar as the reference point in calculating final consumer (supermarket level) meat prices. as well as an annual consumption of 463,000 tons and 687,000 tons of pork and chicken, respectively.

Assuming that import prices of animal products would be 15% higher (due to transportation and intermediation costs and some adjustment for perfect substitutes), the transition from a situation of Chile being a net exporter with a FOB price as reference to a situation of net importer and a CIF reference price would represent a level of losses for the final consumer of approximately US$690 million annually (or about US$36 per capita). Note that this compares with a yearly consumer surplus loss measured in the maize market of US$1,690 million. This last observation emphasizes that, in the Chilean case at least, the deleterious effects of a GM maize import ban would fall in large part on the owners of the fixed assets in the animal products industry. Of course, considering that chicken and pork are not the only products derived from maize (using slightly more than 80% of total maize), the effect on final consumer expenditures would likely be greater, perhaps on the order of 10% to 20%. Again, expenditure changes would represent an upper limit to final consumer surplus loss, which would be lower to the degree that consumers were to shift diets to alternative protein sources with less or perhaps no dependence on maize as a production input.

## 5. Discussion

In this study, we analyze the consequences that a ban on imports of genetically modified maize could have for welfare measures both of Chilean landowners/producers of conventional (non-GM) maize and of users/consumers of maize. Producer and consumer surplus measures are calculated based on the estimation of supply and demand curves in the domestic market for maize. In the hypothetical scenario of self-sufficiency of non-GM maize for feed, maize landowners/farmers would see their producer surplus increase annually by US$1,000 million, while maize users/consumers would see their consumer surplus decrease by more than US$1,600 million. This latter consequence would represent a collapse in the return on investments to the animal-products sector and a significant reduction in the scale of production, with an accompanying fall in employment throughout the production chain beyond the farmgate. The maize consuming sector would have to return to its size of more than two decades ago. Beyond the redistributive effects from maize users to producers, in this scenario there would be a net social loss of more than US$600 million annually.

Furthermore, the magnitude of the impact of a ban on GM maize without external sources of non-GM substitutes raises a question regarding the longer-term viability of the domestic animal products industry. Our estimates are made as if internal maize users could pass-through large price increases of the maize input (on the order of 350%) to the prices facing the final consumers of poultry, pork and other products on the supermarket shelf. But these price-change estimates are based on an extrapolation of the demand curve for maize that is likely well beyond that which would be tolerated by supermarkets and other intermediaries linking animal product wholesalers to the final consumer. Such intermediaries, in an environment of free trade and geographically close, large-scale poultry and pork producers in Argentina, Brazil and elsewhere, would undoubtedly see an opportunity for arbitrage. Domestic production of poultry, pork and other products would likely move entirely or in part to other countries. This would leave a residual demand for conventional, non-GM grain (for silage and some niche products), which would likely erode and perhaps eliminate in the longer term any short-term gain in producer surplus for landowners and maize farmers.

In a scenario in which the Chilean animal-products sector could access imported conventional, non-GM maize at a “reasonable” price of 20% to 50% higher than the current GM maize price, the welfare impacts of a GM ban would be much mitigated. In the case of an increase of 30% in maize prices, domestic maize producer surplus would increase approximately US$63 million annually. Although much less than in the self-sufficiency scenario, this surplus increase would represent a per-hectare increase in net maize annual revenues of approximately US$600, or an increase in the net present value of maize land of US$12,000, using a 5% discount rate. This would represent a substantial incentive to maize landowners/producers to favor GM maize ban, even if they would not reap the full rewards of such a ban under self-sufficiency. Of course, maize users would see their consumer surplus decrease by approximately US$200 million annually, leaving an annual net social loss on the order of US$140 million per year.

How likely would it be to access non-GM maize internationally at a price that would sustain the domestic animal product industry? In the immediate short term that is unlikely, because it would imply having a complex system in place of international scope that could produce, store, and transport the non-GM product, certified and reliable, and with all the derivative markets that facilitate and lower transactions costs in international grain markets. Yet, although such a system is not yet available, there might be a critical mass on the demand side to establish it, if enough governments follow the Mexican plan to ban GM maize. One should be skeptical, however, of that hypothetical. Therefore, although our estimate of a possible 30% or 50% maize price rise for imported non-GM maize is not unreasonable from the perspective of farm costs, it would seem unrealistic (at least in the near term) from the perspective of delivering the certified commodity in large scale from farms in Iowa, USA or Mato Grosso, Brazil to pork producers in Chile. It would be difficult for investors to make the domestic investments required for the industry survival in the longer term in the light of the returns to comparable investments in countries without GM restrictions – for example, in neighboring Brazil or Argentina – from which to supply Chilean consumers of meat.

The possibility to import animal products significantly mitigates the impact of a GM maize ban on final consumers. We estimate an upper limit to consumer surplus loss for animal product consuming households in the range of US$600 to US$800 million annually, the bulk of that loss being in the form of higher prices for pork and poultry due to higher cost of imports relative to domestic costs without a GM ban. The difference between the loss of consumer surplus as measured in the maize market and the loss to final consumers highlights an important aspect of the consequences of a potential GM maize ban in Chile. Much of the welfare loss on the demand side in the maize market would be in the form of a significant decrease in the economic rent being generated by the domestic animal products industry. This rent appears as income flows to the owners of fixed assets linked to the climate, sanitary conditions and other advantages that yield the country’s high productivity in animal products, especially in poultry and pork. And this lost income would be much larger than the income gains enjoyed by landowner and farmers on the supply side of the domestic maize market, generating a net social loss for Chile as a whole.

Finally, we should point out a political-economic implication of this analysis. The study here focuses on the economic welfare implications of a simple prohibition on the import of transgenic maize for three interest groups: maize landowners/producers, maize consumers in the animal products sector, and final consumers of animal products. Certainly, landowners/producers would be in favor of GM restrictions, while the industry would resist. Both groups, relatively small in number, would see concentrated benefits and costs and be motivated to invest in political activity in pursuit of their interests. Final consumers, however, are numerous and harder to organize and would experience diffused costs that would appear at the individual household level to be relatively small. To the degree that government policy might approximate coherence, one might reasonably expect that a GM import ban on maize would be eventually accompanied by the prohibition of other genetically modified products and their derivatives. In the case of animal production, soy and related products are particularly important, because of their significance in animal feed and because the global production of genetically modified soy is widespread. If a ban on the import of GM maize alone puts the animal production industry in Chile at risk, the additional ban on imports of GM soy would likely mean its definite end. Following the logic of an anti-GM policy stance, however, one could easily imagine arguments made by all parties – except final consumers – to extend prohibitions to animal products derived from GM grains. Such a policy would rescue to some degree the asset owners in the domestic animal products industry by shifting almost the full burden of welfare losses of a coherent anti-GM policy to the mass of households as final consumers.

## Appendix

**Table A1.** Supply model: log maize production national consumption.

	(1)	(2)	(3)
VARIABLES	OLS	HAC	GLSR
log maize price, FOB	0.379***	0.379***	0.335**
	(0.150, 0.608)	(0.113, 0.646)	(0.0757, 0.595)
log precipitation	11.29***	11.29***	8.314**
	(4.958, 17.62)	(5.328, 17.25)	(0.741, 15.89)
log precipitation squared	−0.829***	−0.829***	−0.610**
	(−1.308, −0.350)	(−1.278, −0.380)	(−1.177, −0.0427)
Trend	0.0814***	0.0814***	0.0793***
	(0.0640, 0.0988)	(0.0643, 0.0984)	(0.0534, 0.105)
Trend square	−0.00158***	−0.00158***	−0.00155***
	(−0.00200, −0.00116)	(−0.00196, −0.00120)	(−0.00214, −0.000971)
Constant	−27.27**	−27.27***	−16.95
	(−47.98, −6.558)	(−46.27, −8.274)	(−41.56, 7.662)
Observations	43	43	42
R-squared	0.805		0.625

95% confidence intervals in parentheses, *** *p* < .01, ** *p* < .05, * *p* < .1. OLS = Ordinary Least Squares; HAC=Newey-West standard errors robust to heteroscedasticity and autocorrelation; GLSR = Generalized Least Squares (Cochrane-Orcutt) with robust standard errors.

**Table A2.** Supply model: log area and maize yield national consumption.

	log of planted area		log of yield	
	(1)	(2)	(3)	(4)
VARIABLES	OLS	HAC	HAC	IV-HAC
log maize price, FOB	0.473***	0.473***		
	(0.242, 0.704)	(0.222, 0.724)		
log precipitation	7.533**	7.533**	5.165***	6.068***
	(1.144, 13.922)	(1.037, 14.029)	(1.797, 8.533)	(1.998, 10.138)
log precipitation squared	−0.543**	−0.543**	−0.388***	−0.454***
	(−1.026, −0.060)	(−1.030, −0.056)	(−0.640, −0.135)	(−0.758, −0.150)
Trend	0.022**	0.022**	0.063***	0.065***
	(0.005, 0.040)	(0.003, 0.042)	(0.049, 0.078)	(0.053, 0.076)
Trend squared	−0.001***	−0.001***	−0.001***	−0.001***
	(−0.001, −0.000)	(−0.001, −0.000)	(−0.001, −0.001)	(−0.001, −0.001)
log maize planted area			−0.190*	−0.279**
			(−0.410, 0.030)	(−0.554, −0.004)
Constant	−16.844	−16.844	−13.577***	−15.632**
	(−37.748, 4.061)	(−37.925, 4.238)	(−23.188, −3.965)	(−27.536, −3.728)
Observations	43	43	43	43
R-squared	0.609			0.928

95% confidence intervals in parentheses, *** *p* < .01, ** *p* < .05, * *p* < .1. OLS = Ordinary Least Squares; HAC=Newey-West standard errors robust to heteroscedasticity and autocorrelation; IV-HAC = Estimation with instrumental variables using Generalized Moments Method and HAC. The indirect yield-price elasticity, calculated as yield-area elasticity x surface-price elasticity, is −0.116* [−0.236, 0.004]; The composite production-price elasticity, calculated as surface-price elasticity + indirect yield-price elasticity, is 0.353** [0.152, 0.554]

**Table A3.** Supply model: regional log maize total production estimated by SUR.

	(1)	(2)	(3)	(4)
VARIABLES	O’Higgins	Maule	BioBio y Ñuble	RM
log maize prices, FOB	0.080	0.965***	1.079***	0.705***
	(−0.177, 0.337)	(0.583, 1.347)	(0.536, 1.622)	(0.256, 1.154)
Trend	0.113***	0.080***	0.017	0.096***
	(0.094, 0.133)	(0.050, 0.109)	(−0.025, 0.060)	(0.061, 0.130)
Trend squared	−0.003***	−0.001***	0.002***	−0.003***
	(−0.003, −0.002)	(−0.002, −0.001)	(0.001, 0.003)	(−0.004, −0.002)
log precipitation	3.586**	10.340***	26.281**	6.270***
	(0.827, 6.346)	(4.891, 15.789)	(6.067, 46.495)	(1.913, 10.626)
log precipitation squared	−0.277**	−0.756***	−1.786**	−0.497***
	(−0.496, −0.059)	(−1.170, −0.342)	(−3.198, −0.374)	(−0.849, −0.145)
Constant	0.343	−28.992***	−92.786**	−11.997*
	(−8.194, 8.881)	(−46.689, −11.295)	(−164.771, −20.802)	(−25.264, 1.270)
Observations	43	43	43	43
R-squared	0.776	0.800	0.913	0.602

Confidence intervals at the 95% level are given within parentheses, *** *p* < .01, ** *p* < .05, * *p* < .1.

**Table A4.** Supply model: log regional maize hectares estimated by SUR.

	(1)	(2)	(3)	(4)
VARIABLES	O’Higgins	Maule	BioBio y Ñuble	RM
log maize price, FOB	0.124	0.883***	0.799***	0.622***
	(−0.095, 0.344)	(0.500, 1.266)	(0.409, 1.189)	(0.219, 1.024)
Trend	0.060***	0.016	−0.092***	0.045***
	(0.043, 0.077)	(−0.014, 0.046)	(−0.123, −0.061)	(0.014, 0.076)
Trend squared	−0.002***	−0.001*	0.003***	−0.002***
	(−0.002, −0.001)	(−0.001, 0.000)	(0.002, 0.003)	(−0.003, −0.001)
log precipitation	2.871***	5.556**	17.975***	2.255
	(0.807, 4.935)	(0.728, 10.384)	(4.861, 31.089)	(−1.174, 5.685)
log precipitation squared	−0.227***	−0.409**	−1.233***	−0.187
	(−0.391, −0.064)	(−0.776, −0.042)	(−2.150, −0.317)	(−0.463, 0.090)
Constant	1.014	−13.092	−60.134**	−0.227
	(−5.397, 7.424)	(−28.799, 2.616)	(−106.843, −13.425)	(−10.725, 10.272)
Observations	43	43	43	43
R-squared	0.785	0.430	0.833	0.721

Confidence intervals at the 95% level are given within parentheses, *** *p* < .01, ** *p* < .05, * *p* < .1.

**Table A5.** Supply model: log regional maize yield estimated by SUR.

	(1)	(2)	(3)	(4)
VARIABLES	O’Higgins	Maule	BioBio y Ñuble	RM
log hectares maize	−0.045	0.013	0.194**	0.114**
	(−0.198, 0.108)	(−0.078, 0.104)	(0.011, 0.378)	(0.004, 0.223)
log regional precipitation	0.510	5.984***	12.258*	4.002***
	(−1.043, 2.063)	(2.975, 8.993)	(−1.437, 5.953)	(1.825, 6.179)
log region precipitation squared	−0.037	−0.449***	−0.846*	−0.317***
	(−0.160, 0.085)	(−0.676, −0.221)	(−1.799, 0.107)	(−0.493, −0.142)
Trend	0.057***	0.061***	0.123***	0.046***
	(0.044, 0.070)	(0.049, 0.073)	(0.093, 0.153)	(0.032, 0.060)
Trend squared	−0.001***	−0.001***	−0.001***	−0.000***
	(−0.001, −0.001)	(−0.001, −0.000)	(−0.002, −0.001)	(−0.001, −0.000)
Constant	0.402	−19.076***	−46.273*	−12.094***
	(−4.253, 5.057)	(−28.772, −9.380)	(−94.730, 2.184)	(−18.678, −5.510)
Observations	43	43	43	43
R-squared	0.869	0.934	0.938	0.811

95% confidence intervals in parentheses, and asterisks denote p-values: *** *p* < .01, ** *p* < .05, * *p* < .1. As detailed in the notes to Table 1, the indirect yield price elasticity, calculated as yield-area elasticity x surface-price elasticity: O’Higgins 0.007 [−0.026, 0.040]; Maule 0.012 [−0.082, 0.106]; BioBio-Ñuble 0.152 [−0.058, 0.359], and RM 0.131* [0.013, 0.250]. Price-production elasticity: O’Higgins 0.152 [−0.077, 0.381]; Maule 0.895*** [0.529, 1.261]; BioBio-Ñuble 0.927*** [0.455, 1.399], and RM 0.742*** [0.294, 1.191].
